# Self-Deceived Individuals Are Better at Deceiving Others

**DOI:** 10.1371/journal.pone.0104562

**Published:** 2014-08-27

**Authors:** Shakti Lamba, Vivek Nityananda

**Affiliations:** 1 Centre for Ecology and Conservation, College of Life and Environmental Sciences, University of Exeter, Penryn Campus, Cornwall, United Kingdom; 2 Department of Anthropology, University College London, London, United Kingdom; 3 Biological and Experimental Psychology, School of Biological and Chemical Sciences, Queen Mary University of London, London, United Kingdom; 4 Institute of Neuroscience, Henry Wellcome Building for Neuroecology, Newcastle University, Framlington Place, Newcastle upon Tyne, United Kingdom; University of Freiburg, Germany

## Abstract

Self-deception is widespread in humans even though it can lead to disastrous consequences such as airplane crashes and financial meltdowns. Why is this potentially harmful trait so common? A controversial theory proposes that self-deception evolved to facilitate the deception of others. We test this hypothesis in the real world and find support for it: Overconfident individuals are overrated by observers and underconfident individuals are judged by observers to be worse than they actually are. Our findings suggest that people may not always reward the more accomplished individual but rather the more self-deceived. Moreover, if overconfident individuals are more likely to be risk-prone then by promoting them we may be creating institutions, including banks and armies, which are more vulnerable to risk. Our results reveal practical solutions for assessing individuals that circumvent the influence of self-deception and can be implemented in a range of organizations including educational institutions.

## Introduction

Self-deception - individuals' false beliefs about their abilities – is widespread in humans. People consistently overrate their capabilities [1], [2], suffer from positive illusions [3] and deny their disabilities [4]. We are remarkably prone to both overconfidence - reflecting inflated beliefs about our abilities - and underconfidence arising from a negative self-image [5]–[7]. These biased beliefs can lead to costly errors with disastrous consequences including airplane crashes, financial meltdowns and war [5], [6], [8], [9]. Why is this potentially harmful trait so common? A controversial theory proposes that self-deception has evolved to facilitate the deception of others [5], [6], [8], [10]. Self-deceived individuals may be less likely to produce cues, such as stress, that reveal deception [5]. Here, we provide the first direct test of this hypothesis in a real-world setting and find support for it. We demonstrate that individuals who overestimate their abilities at a task are overrated at that task by observers. Equally, individuals who falsely believe that they are not good at the task are judged by observers to be worse at it than they actually are. Our findings suggest that people may not always reward the more accomplished individual but rather the more self-deceived. Moreover, if overconfident individuals are more likely to be risk-prone [11] then by promoting such individuals we may be creating institutions, including banks, trading floors, emergency services and armies, that are also more vulnerable to risk.

Many authors argue that the intra-personal gains of positive self-deception provide an adequate account for its prevalence [3], [12]. For example, positive beliefs about oneself are associated with increased well-being and enhanced status [3], [13], [14]. Overconfidence may also be advantageous when competitors are uncertain about their relative abilities [15]. An alternative theory suggests that self-deception first evolved in the context of inter-personal relations because it facilitates the deception of others by eliminating cues that reveal deception [5], [6], [8], [10]. According to this view, the intrapersonal advantages of self-deception are a by-product rather than the driving force for the evolution of this trait. While this idea has theoretical traction [16], it remains empirically untested. We present the first direct evidence suggesting that fooling oneself helps fool others.

Our study was conducted within the context of the tutorial system implemented at some universities, where students meet in small groups on a weekly basis to review, debate and discuss course material with a tutor and each other. In these tutorials, students interact freely with each other and the tutor. At the end of the first tutorials for a first-year undergraduate course held in the first term, students were asked to privately predict the performance of each of their peers from the tutorial group; they were asked to predict the absolute grade and relative rank they thought each of their classmates would obtain for the next assignment that they would complete for the course. Similarly, they assessed their own performance. Participants received one British pound for each correct prediction that they made. 71 out of 73 participants did not know anyone in their tutorial group prior to enrolling at university only 3 weeks before the first tutorial was held. They were thus limited to basing their predictions solely on their interactions in a single tutorial. We later obtained participants' actual grades from the lecturer for the course. All assignments were marked double-blind, i.e. the lecturer did not know the identity of the students while grading them.

We measure self-deception as the difference between the self-estimate and the actual grade of an individual. We measure deception as the difference between the median estimate made by peers and the actual grade of an individual. We measure the susceptibility to being deceived as the median of the difference between the grades that an individual predicted for peers and the actual grades that those peers received from the tutor. Table 1 provides a summary of these behavioural measures and how they are calculated.

**Table 1:** Summary of behavioural measures.

Behaviour of focal individual	Description	Calculation^1^
Self-deception	Self-estimate of grade – Actual grade received	s-a_self_
Deception	Median estimate by peers - Actual grade received	Median (p_i_)−a_self_
Susceptibility to being deceived	Median (Grades predicted for peers – Actual grades received by peers)	Median {o_i_−(a_other_)_i_}

1 s = grade predicted by focal individual for self; a_self_ = actual grade received by focal individual; p = grade predicted by peer for focal individual; o = grade predicted by focal individual for peer; a_other_ = actual grade received by peer; i = individual in tutorial group other than self.

If self-deception facilitates deception then we expect the measures of self-deception and deception to be positively associated with each other. Concurrently, if self-deception diminishes individuals' ability to detect deception by others then we expect our measure of self-deception to be positively associated with the susceptibility to being deceived.

The study was run at two universities in London, University College London and Queen Mary University of London, and the average number of students in each tutorial group was about 8. Table 2 presents summary statistics for the tutorial groups included in our study and Table 3 provides a demographic description of our study sample. The study was run two times, once at the end of the first tutorial and a second time at the end of a tutorial about six weeks later to test whether the association between self-deception and deception weakened with extended interaction between participants.

**Table 2:** Sample sizes and sex ratios for the twelve tutorial groups included in this study.

Tutorial group	University	Number of participants^2^	
		Week one	Week six
1	UCL	3 (9)	3 (8)
2	UCL	2 (8)	0 (7)
3	UCL	7 (7)	3 (6)
4	UCL	6 (7)	5 (8)
5	UCL	8 (8)	9 (9)
6	UCL	3 (10)	0 (9)
7	QMUL	8 (8)	7 (7)
8	QMUL	5 (6)	5 (6)
9	QMUL	7 (7)	7 (7)
10	QMUL	7 (7)	8 (8)
11	QMUL	8 (9)	6 (6)
12	QMUL	9 (10)	6 (9)

1 UCL - University College London; QMUL - Queen Mary University of London.

2 Numbers in parentheses indicate the total number of students present during the tutorial since not all students chose to participate in this study.

29 students participated from UCL^1^ and 44 students from QMUL^1^ (total n = 73). The mean age ± s.d. of participants was 18.76±0.90 years and 85% were female.

**Table 3:** Summary of demographic variables of study participants.

University	Age ± s.d.	Percentage female	Ethnicity (% white)	Family income (GBP)*± s.d.
UCL	18.97±0.98	77	71	61,920±17,515
QMUL	18.60±0.82	91	37	69,208±18,178

*Participants' parent's professions were assigned to categories specified by the Office of National Statistics (ONS). Occupational data from the ONS were used as a reference and the gender-specific median annual full-time London earnings of the relevant category were assigned to each parent. The earnings of both parents were added together to obtain the family income. For the partial correlation analyses, the Hollingshead four factor index [20] was derived from individual earnings (as above) for each parent and the index for the parent with the highest earnings was used as a measure of family income. The ONS table used to calculate family income was titled: PROV - Work Region Occupation SOC10 (2) Table 3.7a Annual pay - Gross 2011.

## Results and Discussion

### 1. Is self-deception about one's ability associated with how deceived others are about one's ability?

**Figure 1:**
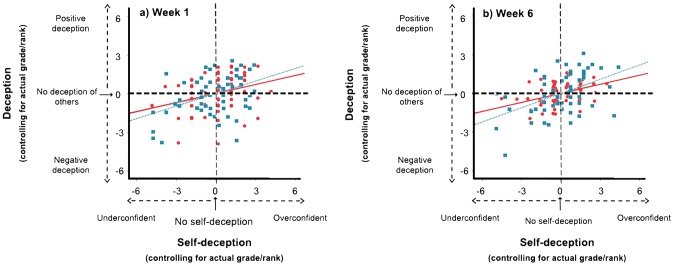
Self-deception and deception of others are positively associated. Scatterplots with best-fit lines for residuals of deception (median estimate of focal individual's performance by peers – focal individual's actual performance) plotted against residuals of self-deception (self-estimate of focal individual's performance – focal individual's actual performance) based on absolute grades (red circles and red bold lines) and relative ranks (blue squares and blue dotted lines) in (a) week one and (b) week six. The residuals were obtained via a partial correlation analysis that regressed (i) self-deception against actual grade and (ii) deception against actual grade. Mean ± s.d. of absolute level of self-deception was 1.93±1.54 grades and 2.11±1.70 ranks in week one and 1.72±1.42 grades and 2.04±1.99 ranks in week six. Mean ± s.d. of absolute level of deception was 1.90±1.48 grades and 1.80±1.30 ranks in week one, and 1.27±1.03 grades and 1.86±1.59 ranks in week six.

Individuals who rated themselves higher were rated higher by others, irrespective of their actual performance. There is a significant positive correlation between the measures of self-deception and deception based on both absolute grades and relative ranks, after controlling for actual grade and rank respectively (Grades: partial correlation coefficient = 0.31, one tailed p = 0.01, df = 69; Ranks: partial correlation coefficient = 0.42, one-tailed p<0.001, df = 63; Figure 1a). The significant positive relationship between self-deception and deception is unaffected by an individual's sex, age, family income, tutorial group size or university.

### 2. Does extended interaction with individuals diminish a self-deceived individual's ability to deceive those individuals?

Extended interaction may diminish or eliminate a self-deceived individual's ability to deceive another individual. This is because deception only works as long as the deceived individual has incomplete information about the deceiver and extended interaction is likely to provide the deceived individual with more information about the deceiver's true abilities. We therefore repeated the exercise at the end of a tutorial about six weeks later to investigate whether the association between self-deception and deception weakened with extended interaction between participants. We find that the measures of self-deception and deception remain significantly correlated (Grades: partial correlation coefficient = 0.40, one-tailed p = 0.001, df = 57; Ranks: partial correlation coefficient = 0.47, one-tailed p<0.001, df = 51; Figure 1b) suggesting that there was little effect of interaction on this timescale. It is worth noting that at one of the universities, levels of self-deception changed significantly in week six compared to week one (Wilcoxon signed ranks test Z = −3.311, n = 18, p = 0.001). Since the association between self-deception and deception remains intact in week six, together these results suggest that as individuals' levels of self-deception change, their peers' judgements of them also change.

### 3. Is the degree to which individuals are self-deceived constrained by how believable their self-deception is to others?

Two factors are likely to constrain the degree to which individuals are self-deceived. First, the extent to which individuals' self-deception is believed by others, and second, the amount of error and risk it exposes them to. Self-deception is therefore expected to be anchored by an individual's actual capabilities to represent “believable deviations from reality” [5]. For instance, a B-grader should be more likely to believe that she will get an A or a C grade than an E grade. Similarly, a D-grader should be more likely to believe that she will get a C or an E grade than an A grade. In other words, we should observe a positive correlation between participants' self-predictions and their actual performance. We find that self-prediction and actual performance show no correlation based on absolute grades (Week 1 - Spearman rank correlation coefficient = 0.03, one-tailed p = 0.40, n = 72; Figure S1a) but a significant positive correlation based on relative ranks (Week 1 - Spearman rank correlation coefficient = 0.39, one-tailed p = 0.001, n = 66; Figure S1a). Concurrently, we find that individuals' peers' predictions about them do not correlate with their actual performance based on absolute grades but do so based on relative ranks (Week 1 - Grades: Spearman rank correlation coefficient = −0.01, one-tailed p = 0.46, n = 73; Ranks: Spearman rank correlation coefficient = 0.39, one-tailed p<0.001, n = 73; Figure S1b). Figure S2 displays results for week six.

The above results suggest that self-deception may be anchored by actual performance only when individuals evaluate themselves within a relative framework (ranks) and not in an absolute framework (grades). The finding that peers' predictions are only anchored around an individual's actual performance when her self-predictions are too, further supports the idea that an individual's beliefs about herself influenced her peers' impressions of her. Moreover, it suggests that self-deception is believed by others even if it is not anchored around real ability. Thus, exposure to risk and error may be the more important constraint on levels of self-deception than how believable it is to others.

### 4. Are individuals who are self-deceived poor at detecting deception by others?

There may be several ways to detect deception by others such as relying on bodily cues and signals of deception (e.g. pitch of voice, fidgeting [6], [17]). One could also infer deception based on knowledge of the state of the world. In the latter case, we need to compare our own knowledge of the state of the world to the one that is being presented to us by the deceiver. Holding an erroneous representation of the state of the world may, therefore, interfere with our ability to detect deception. Since self-deception involves holding inaccurate beliefs about our abilities and the state of the world it may consequently diminish our ability to detect deception by others; in other words, it may make us more susceptible to being deceived.

**Figure 2:**
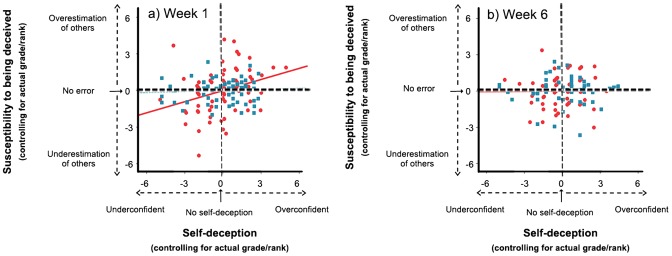
Self-deception and susceptibility to being deceived are positively associated. Scatterplots with best-fit lines for residuals of susceptibility to being deceived (median of the difference between a focal individual's estimate of peer performance and the actual performance of peers) plotted against residuals of self-deception (self-estimate of focal individual's performance – focal individual's actual performance) based on absolute grades (red circles and red bold lines) and relative ranks (blue squares and blue dotted lines) in (a) week one and (b) week six. The residuals were obtained via a partial correlation analysis that regressed (i) self-deception against actual grade and (ii) susceptibility to being deceived against actual grade.

We measured individuals' susceptibility to being deceived as the median difference between the grades/ranks that they predicted for peers and the actual grades/ranks that the peers received from the tutor. In week one there is a significant positive correlation between the measures of self-deception and susceptibility to being deceived based on absolute grades but not based on relative ranks, after controlling for actual grade and rank respectively (Grades: partial correlation coefficient = 0.302, one tailed p = 0.01, df = 70; Ranks: partial correlation coefficient = 0.066, one-tailed p = 0.30, df = 63; Figure 2a). Thus, overconfident individuals tended to overestimate the abilities of others while underconfident individuals underestimated the abilities of others. However, in week six, self-deception is no longer correlated with susceptibility to being deceived based on grades or ranks (Grades: partial correlation coefficient = 0.008, one tailed p = 0.48, df = 57; Ranks: partial correlation coefficient = −0.01, one-tailed p = 0.47, df = 51; Figure 2b).

Our results suggest the possibility that self-deception diminishes an individual's ability to accurately estimate the abilities of others when they use an absolute criterion (grades) to do so, but not when they use a relative criterion (ranks). Hence, another important cost of (and therefore constraint on) being self-deceived may be an impaired ability to detect deception by others. However, we also find that the association between self-deception and the susceptibility to being deceived disappears with extended interaction, perhaps because individuals gather more information about their peers and become less prone to being deceived. This is supported by the finding that the absolute level of deception based on grades is significantly lower in week six compared to week one (Wilcoxon signed rank test Z = −2.94, n = 58, p = 0.003)

### Conclusion

Our results support the idea that self-deception facilitates the deception of others. Overconfident individuals were overrated and underconfident individuals were underrated. While the benefits of being overconfident are apparent, it is less clear whether underconfidence can also be advantageous. There may, however, be situations in everyday life where individuals underplay their abilities to their competitors in order to either avoid immediate conflict or to steal an advantage at the right moment, the “underdog” effect. “Dummying up” or appearing less knowledgeable than you are may also be a way to avoid working as hard as others (pg 167 in [6]).

Since students hardly knew each other, they had little information about what the other members of their tutorial group thought about their academic abilities and we did not tell them the predictions their peers made for them. Thus, their peers' ratings of them could not have influenced their ratings of themselves. The study was conducted among Psychology and Anthropology students, both programmes with higher female enrolment, making our study sample female-biased. While we find no effect of sex on the relationship between self-deception and deception, previous studies have found that men are more likely to be overconfident and women are more likely to be the opposite [18]. It is therefore notable that overconfident women are equally likely to create a false positive impression on observers as overconfident men.

On a practical level, we find that a relative framework of evaluation (e.g. ranks) may be superior to an absolute framework (e.g. grades) in terms of individuals' ability to both evaluate themselves and others. Individuals' evaluations of themselves are anchored around reality when they use ranks but not when they use grades (Results and Discussion Section 3). Concurrently, their estimations of others' abilities are unaffected by their own self-deception when using ranks but not when using grades (Results and Discussion Section 4). This may simply be because ranking individuals is a computationally easier exercise than predicting grades since each individual can only be assigned a unique rank but can be assigned any of a set of grades. Alternatively, directly comparing individuals with each other may allow people to form more accurate evaluations of their abilities compared to when they evaluate them in isolation. Our results also advocate the use of double-blind assessment wherever possible, such as in educational establishments and the scientific peer-review system, in order to circumvent the influence of self-deception by the assessee on the assessor.

Our findings have implications for many types of social interactions but especially for those involving partner-choice (e.g. choosing mates, hiring people for jobs), suggesting that we may be rewarding overconfidence and penalizing underconfidence irrespective of an individual's capability. Furthermore, if overconfident individuals are more likely to be risk-prone [11] then by promoting such individuals we may be creating institutions such as banks, trading floors and armies, that are also more vulnerable to risk. From our smallest interactions to the institutions we build, self-deception may play a profound role in shaping the world we inhabit.

## Materials and Methods

This study has approval from the Ethics Committees at University College London (UCL) and Queen Mary University of London (QMUL). Informed consent was obtained from all participants.

### Study set-up

The tutor in charge conducted the tutorials and students were unaware that they would be requested to participate in our study during the tutorials. We entered the tutorial room once the tutor had finished the tutorial. Students were informed that we were conducting a study on people's ability to evaluate themselves and their peers but the precise research question and hypothesis being tested were not disclosed. All students were then provided an information sheet (see Information Sheet S1) and students who did not want to participate in our study were allowed to leave. We then handed out nametags to the participating students (so that they could clearly identify each other) and an evaluation sheet on which they recorded the absolute grades and relative ranks that they expected each of the participants in their tutorial group (including themselves) to receive for the next assignment that they completed for the course. Participants were instructed not to predict the grades and ranks of members of their tutorial who had declined to participate in this study.

Participants were not informed which predictions were correct and were paid £1 for each grade or rank that they predicted correctly. Participants were informed about their earnings from both tutorials and all payments were made only after all data collection was complete. Participants were only paid for a prediction if it was exactly correct and not paid based on how close the predicted grade/rank was to the actual grade/rank, thus incentivizing individuals to be as accurate as possible in their predictions.

### Analyses

We obtained the partial correlation between self-deception and deception as well as self-deception and susceptibility to being deceived controlling for actual grades/ranks. We repeated these analyses controlling for age, sex, family income, tutorial group size and university. Non-parametric statistics were used to analyse the overall correlation between self and other predictions and actual performance. All analyses were run in SPSS version 20.0.0 [19].

## Supporting Information

Figure S1 **Correlations between self and peer predictions and actual performance in week one.** Scatterplots with best-fit lines for a) self-predictions and b) peer predictions plotted against actual performance based on absolute grades (red circles and red bold lines) and relative ranks (blue squares and blue dotted lines) in week 1.
(TIF)

Figure S2 **Correlations between self and peer predictions and actual performance in week six.** Scatterplots with best-fit lines for peer predictions plotted against actual performance Scatterplots with best-fit lines for a) self-predictions and b) peer predictions plotted against actual performance based on absolute grades (red circles and red bold lines) and relative ranks (blue squares and blue dotted lines) in week six.
(TIF)

Data File S1 **Grades and ranks predicted by participants during tutorials in week one and week six, actual grades and ranks obtained in the subsequent assignment and calculated measures of self-deception and deception based on these grades and ranks.**
(XLSX)

Information Sheet S1 **Information and evaluation sheets provided to participants during the experiment.**
(DOCX)
