# Supplementary figures and images for: Self-Deceived Individuals Are Better at Deceiving Others
Source: PLoS One. 2014 Aug 27;9(8):e104562. doi: 10.1371/journal.pone.0104562 (PMC4146531; doi:10.1371/journal.pone.0104562)

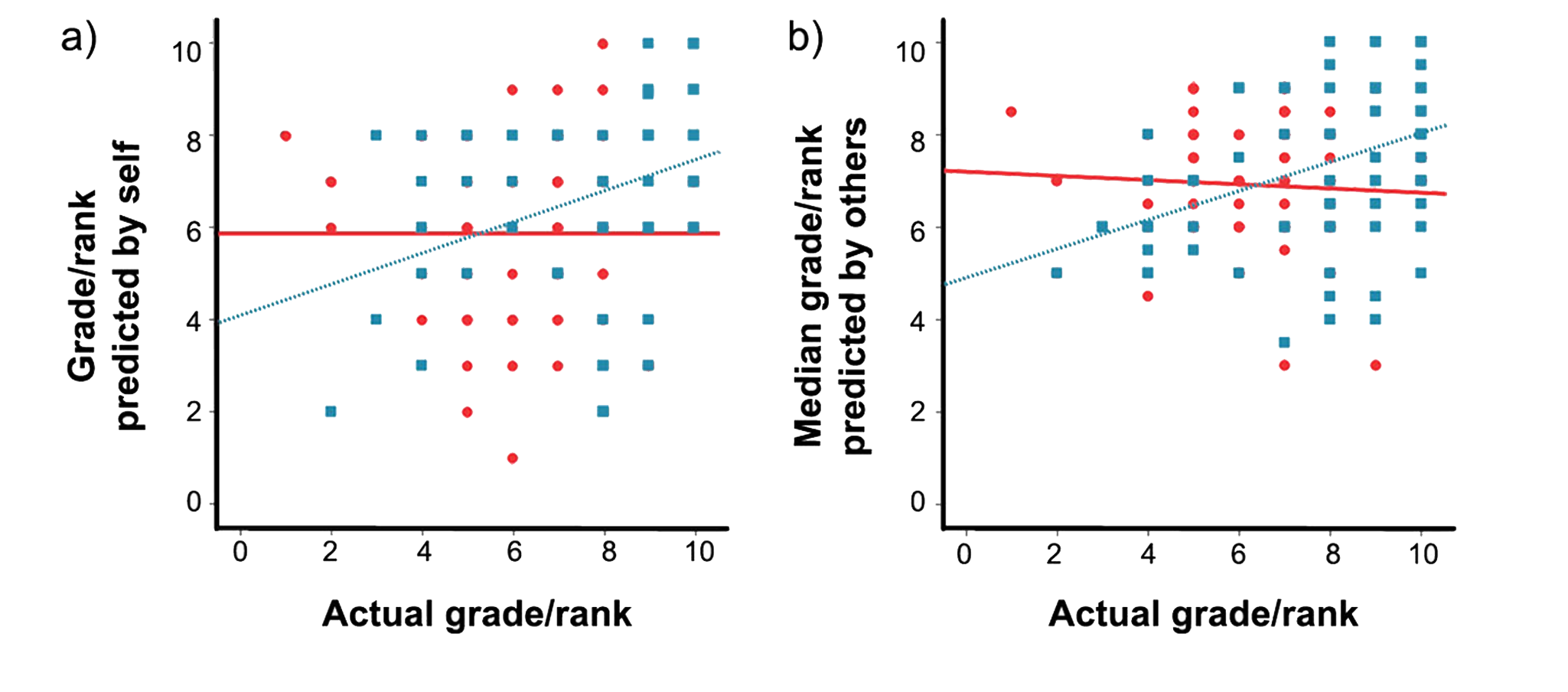

Supplement: Figure S1 — Correlations between self and peer predictions and actual performance in week one. Scatterplots with best-fit lines for a) self-predictions and b) peer predictions plotted against actual performance based on absolute grades (red circles and red bold lines) and relative ranks (blue squares and blue dotted lines) in week 1. (TIF) [file pone.0104562.s001.tif]

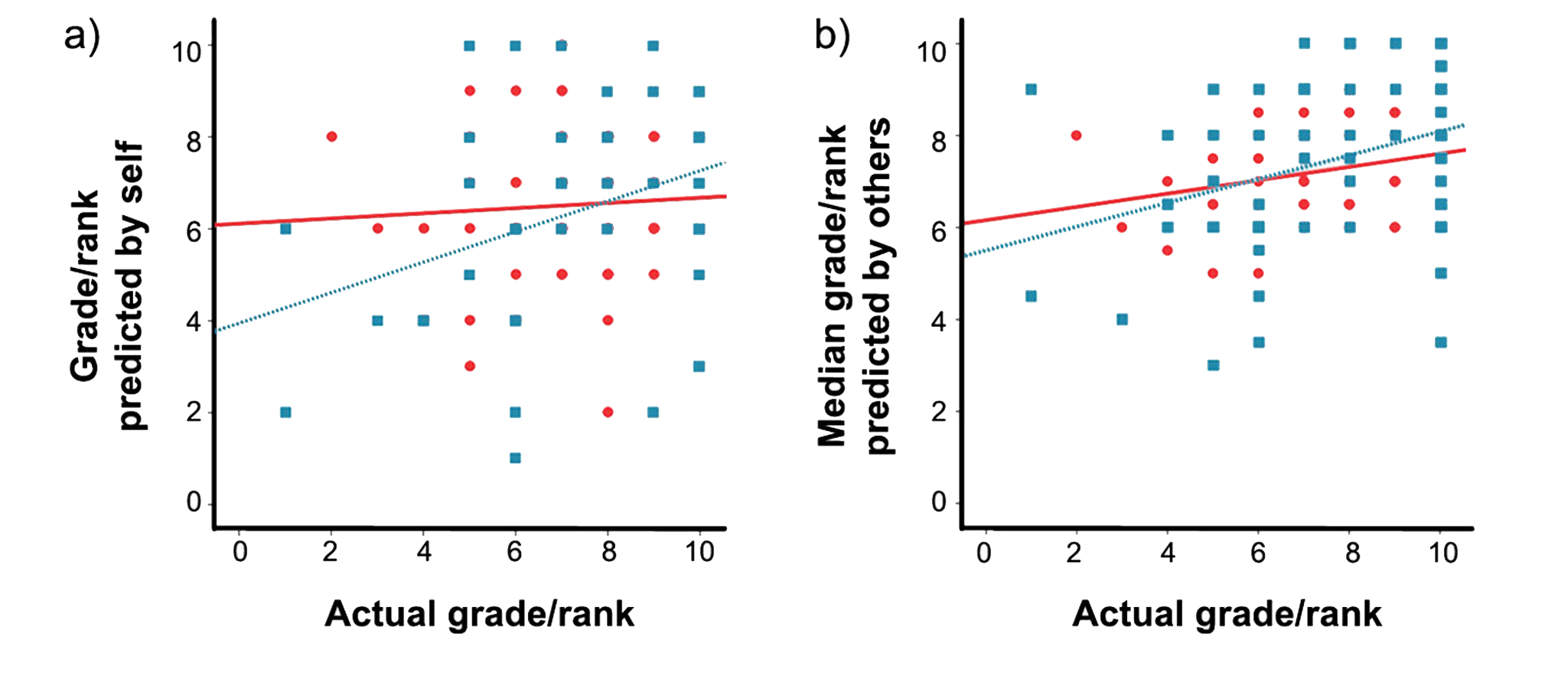

Supplement: Figure S2 — Correlations between self and peer predictions and actual performance in week six. Scatterplots with best-fit lines for peer predictions plotted against actual performance Scatterplots with best-fit lines for a) self-predictions and b) peer predictions plotted against actual performance based on absolute grades (red circles and red bold lines) and relative ranks (blue squares and blue dotted lines) in week six. (TIF) [file pone.0104562.s002.tif]
