# Supplementary material for: Self-Deceived Individuals Are Better at Deceiving Others
Source: PLoS One. 2014 Aug 27;9(8):e104562. doi: 10.1371/journal.pone.0104562 (PMC4146531; doi:10.1371/journal.pone.0104562)
Supplement: Information Sheet S1 — Information and evaluation sheets provided to participants during the experiment. (DOCX) [file pone.0104562.s004.docx]

**Supplementary Materials**

Information and evaluation sheets

Figures S1-S2

## 1. Information and evaluation sheets

### 1.1 Information sheet

Hi. We are conducting a study on people’s ability to evaluate themselves and their peers. We would be happy if you could spare the time to participate.

***1. What do I have to do?***

If you decide to participate, you will have to do the following:

1. **You will have to predict the grade that you expect to receive for the next assignment (essay/ exercise etc) that you will complete as a requirement for this course.**

We do not want you to predict the overall grade that you expect to receive for this course. We want you to specifically predict the grade you expect to receive for the next assignment that you will complete for this course. Similarly, you will also have to predict the rank you expect to receive relative to the rest of the members of your tutorial group who are participating in this study.

1. **You will have to predict the grades that you expect each of the other members of your tutorial group (who have also agreed to participate in this study) to receive for the next assignment (essay/ exercise etc) that they will complete as a requirement for this course.**

Again, we do not want you to predict the overall grade that you think they will receive for this course. We want you to specifically predict the grade you expect them to receive for the next assignment that they will complete for this course. Similarly, you will also have to predict the rank you expect them to receive relative to the rest of the members of your tutorial group who are participating in this study (including you).

1. You will have to allow us, the researchers, to access the actual grade and rank that you obtain for the next assignment that you will complete as well as the aggregate grade that you obtain for the course from the course organiser for this course. **Note that the actual grade and rank that you receive for the assignment you have just completed or any other assignment that you complete for this course will not in any way be affected by whether you decide to participate in this study or not. In fact your assignments will be marked blindly such that even the tutor marking your assignments will not know who’s assignment s/he will be marking.**

In addition you will be required to tell us your age, gender and some basic information about your educational background. Once you have filled in the response sheet, you will put this sheet in the envelope provided, seal the envelope, and hand it over to the researchers.

***2. Who will have access to this information?***

- We guarantee that your personal details, the grade/rank you predict for yourself, the grades/rank you predict for others and the actual grade/rank that you receive for the assignment that you have just completed will remain completely private.
- **This information will not be available to anyone other than us, the researchers conducting the study. Hence, neither the course organisers, nor the other students taking the course nor anyone else will have access to your predictions.**
- Moreover, all data will be stored in our database in an anonymised format such that each individual will be assigned a unique id code and no participant will be individually identifiable in the database.

***3. What’s in it for me?***

- You will earn real money in this study based on the accuracy of your predictions.
  - Once we, the researchers, know your actual grade/rank we will check whether the grade/rank you predicted for yourself is the same as the actual grade/rank that you received for the assignment that you have just completed.
    - Similarly, once we know the actual grades/ranks of each of the other members of your tutorial group (who have also agreed to participate in this study), we will check whether the grade/rank you predicted for each of these other members of your tutorial group is the same as the actual grade/rank that they received for the assignment that you have all just completed.
    - You will receive **£1 for every correct prediction that you make**. For example, if you accurately predict your own grade/rank as well as the grades/ranks of say six other people, you will receive a total of £7. However, you will only get paid for a prediction if it is exactly correct. In other words, you will not get paid for a prediction based on how close the predicted grade/rank is to the actual grade/rank. You will not be told which of your predictions were correct. You will be paid £1 if you predict either the grade or rank correctly.
    - **Exception:** In the event that you accurately predict the grades/ranks of all the members of your tutorial group. In this exceptional case, you will receive £1 for all except one of your predictions. We will randomly select the prediction for which you will not be paid. You will not be told which of your predictions you are being paid for. Thus, people who accurately predict the grades/ranks of all except one member in their tutorial group will be paid the same as those who accurately predict the grades/ranks of all the members in their tutorial group. Therefore, since you will never know whether all your predictions were correct or not you cannot know the grades/ranks of the other members of your group on this basis. Similarly, the other members of your group will never know your grades/ranks on this basis.
      - Your earnings from this study will be given to you next term.

***4. Do I have to participate in this study?***

No. Don’t worry if you do not wish to participate in this study. Just return this sheet unfilled to the researcher and feel free to leave the room. Remember that the actual grades/ranks that you receive for the next assignment that you will complete or any other assignment that you complete for this or other courses will not in any way be affected by whether you decide to participate in this study or not. If you do decide to participate in this study, remember that you are free to withdraw from the study at any point.

If you are happy to participate in this study please sign the attached consent sheet.

Thank you for your time.

Shakti Lamba and Vivek Nityananda

### 1.2 Evaluation sheet

**1. Personal details**

1.1 Your name:

1.2 Your current university:

1.3 Name of this course:

1.4 Your gender (male/female):

1.5 Your age:

1.6 Your nationality:

1.7 Your ethnicity:

1.8 Main subject/s of the degree you are currently undertaking:

1.9 Main subject/s of any previous degrees you have undertaken:

1.10 Highest level degree (e.g. Highschool A levels, Bachelor’s, Masters etc.) held:

1.11 Number of years of **post-school** education that you have undertaken (indicate none if this is your first undergraduate degree):

1.12 Father’s current or most recent occupation:

1.13 Mother’s current or most recent occupation:

## 2. Self and peer prediction

For each individual listed below, **including yourself**, please record the grade you expect them to receive for their next assignment for this course. The grading scheme is indicated in the table so please put a tick in the appropriate box for each listed individual. In addition please indicate what rank you expect them to receive relative to the other members of your tutorial group who are participating in this study (including you).

| 2.1 | 2.2 | | | | | | | | | | | | | 2.3 | 2.4 | 2.5 | 2.6 |
| --- | --- | --- | --- | --- | --- | --- | --- | --- | --- | --- | --- | --- | --- | --- | --- | --- | --- |
| Name of student | Predicted letter grade (put a tick in the appropriate box for each listed individual) | | | | | | | | | | | | | Predicted rank | How long have you known this person?  (in weeks) | Have you previously taken an academic course with this person?  yes/no | Have you studied with this person prior to enrolling at this university? yes/no |
|  | A+ | A | A- | B+ | B | B- | C+ | C | C- | D+ | D | D- | F |  |  |  |  |
|  |  |  |  |  |  |  |  |  |  |  |  |  |  |  |  |  |  |
|  |  |  |  |  |  |  |  |  |  |  |  |  |  |  |  |  |  |
|  |  |  |  |  |  |  |  |  |  |  |  |  |  |  |  |  |  |
|  |  |  |  |  |  |  |  |  |  |  |  |  |  |  |  |  |  |
|  |  |  |  |  |  |  |  |  |  |  |  |  |  |  |  |  |  |
|  |  |  |  |  |  |  |  |  |  |  |  |  |  |  |  |  |  |
|  |  |  |  |  |  |  |  |  |  |  |  |  |  |  |  |  |  |
|  |  |  |  |  |  |  |  |  |  |  |  |  |  |  |  |  |  |

In case there are more students in your class than there is space provided for on this sheet, please fill in the same details for the other students on another sheet of paper and attach it to this form.

## 3. Supplementary Figure Legends

**Figure S1.**

Correlations between self and peer predictions and actual performance in week 1. Scatterplots with best-fit lines for a) self-predictions and b) peer predictions plotted against actual performance based on absolute grades (red circles and red bold lines) and relative ranks (blue squares and blue dotted lines) in week 1.

**Figure S2**

Correlations between self and peer predictions and actual performance in week 6. Scatterplots with best-fit lines for peer predictions plotted against actual performance Scatterplots with best-fit lines for a) self-predictions and b) peer predictions plotted against actual performance based on absolute grades (red circles and red bold lines) and relative ranks (blue squares and blue dotted lines) in week 6.
